# Altered profile of circulating endothelial progenitor cells in obstructive sleep apnea

**DOI:** 10.1007/s11325-012-0781-4

**Published:** 2012-11-21

**Authors:** Macy Mei-Sze Lui, Hung-Fat Tse, Judith Choi-Wo Mak, Jamie Chung-Mei Lam, David Chi-Leung Lam, Kathryn C. B. Tan, Mary Sau-Man Ip

**Affiliations:** Department of Medicine, Queen Mary Hospital, The University of Hong Kong, Pokfulam, Hong Kong SAR, China

**Keywords:** Advanced glycation end-products, Endothelial progenitor cells, Intermittent hypoxia

## Abstract

**Background:**

Obstructive sleep apnea (OSA) is independently associated with endothelial dysfunction, which may be perpetuated by alteration in endothelial repair capacity. Our study evaluates changes in endothelial progenitor cell (EPC) profile in relation to OSA and the role of advanced glycation end-products (AGE) in this relationship.

**Methods:**

Consecutive Chinese adults undergoing sleep studies, who had no medical illnesses or regular medications, were enrolled. Subjects with morbid obesity or grossly elevated lipoprotein levels were excluded from analysis. Circulating EPC was measured with flow cytometry analysis.

**Results:**

Seventy-two subjects, 64 % with OSA defined by apnea–hypopnea index (AHI) ≥ 5, were analyzed. CD34+ cell counts were positively correlated with oxygen desaturation index (ODI) (*r* = 0.250, *p* = 0.041) and duration of oxygen desaturation <90 % (T90) (*r* = 0.261, *p* = 0.033) and negatively with minimal oxygen saturation (*r* = −0.247, *p* = 0.044) after adjusting for age, glucose, body weight, and smoking status. AGE was positively correlated with indices of OSA severity (AHI, *r* = 0.249, *p* = 0.042; ODI, *r* = 0.244, *p* = 0.047; T90, *r* = 0.243, *p* = 0.047; minimal oxygen saturation, *r* = −0.251, *p* = 0.041) and negatively with CD133+ cells (*r* = −0.281, *p* = 0.021). On stepwise multiple linear regression analysis, minimal oxygen saturation (*p* = 0.013) and CD133+ cell counts (*p* = 0.029) were found to be significant determinants of AGE level (*R* ^2^ = 0.147).

**Conclusions:**

Nocturnal hypoxemia in OSA subjects was associated with increase in endothelial cells (CD34+) which may promote vascular repair. Accumulation of AGE in OSA may lead to diminution in early EPC (CD133+) and endothelial repair capacity over time, thus contributing to vascular pathogenesis.

## Introduction

Obstructive sleep apnea (OSA) has been shown to predispose to atherosclerotic vasculopathy including coronary artery disease and cerebrovascular accidents and result in increased mortality [1, 2]. The presence of OSA in healthy subjects without conventional cardiovascular risk factors was associated independently with early signs of atherosclerosis as indicated by carotid-media-intimal thickness [3]. Current knowledge of atherosclerosis suggests that formation of atherosclerotic plagues is preceded by a series of prestructural pathologic events including endothelial dysfunction, vascular inflammation, and oxidative stress, which involve complex interplay of many cells and molecules. One such biochemical mediators, namely advanced glycation end-products (AGE), play a pivotal role in promoting atherosclerosis, especially in the setting of insulin resistance [4]. AGE is generated by nonenzymatic glycation and oxidation of proteins and lipids, in states of heightened oxidative stress and hyperglycemia [5]. AGE triggers a number of cellular cascades that promote endothelial dysfunction and atherosclerosis, including deleterious effects on endothelial progenitor cells (EPC) [6]. We have previously reported that OSA was associated with elevated AGE independent of diabetes or insulin sensitivity [7, 8], and this could be reduced with CPAP treatment [9], suggesting that accumulation of AGE may play a role in vascular pathogenesis in OSA.

Endothelial function, often measured noninvasively by endothelial dependent vasodilatation in response to reactive hyperemia, is convincingly shown to be impaired in OSA independent of other risk factors [10, 11]. The recent discovery of EPC has revolutionized the horizons of regenerative cardiovascular medicine. EPC is believed to reflect another aspect of endothelial function and refers to the group of circulating cells that mediates endothelial repair at sites of vascular injury directly by differentiation into mature endothelial cells, or indirectly by secreting vaso-active substances promoting repair or mediating angiogenesis at sites of ischemia [12, 13]. Understanding EPC biology in OSA would contribute to the knowledge of vascular pathogenesis in OSA, as well as potential therapeutic interventions to treat incident diseases [14]. Several previous studies have investigated the relationship between OSA and EPC, but results were inconsistent and most were limited by sample size and presence of confounding factors. In the present study, we hypothesized that OSA may alter EPC profile, and this could be related to the accumulation of AGE.

## Methods

### Subjects and protocol

Consecutive healthy adults undergoing overnight polysomnography (PSG) at the Ho Ting Shek Sleep Disorders Center, Queen Mary Hospital, have been prospectively recruited since June 2008 in a study to explore the relation between endothelial dysfunction, circulatory biomarkers of oxidative stress, inflammation, and vascular remodeling in OSA. In the current study which focuses on EPC and AGE, subjects were recruited between October 2008 and March 2011. Exclusion criteria were history of hypertension, diabetes mellitus or hyperlipidemia on medications, atherosclerotic vascular diseases, and other chronic medical diseases; newly detected high blood pressure with systolic blood pressure >160 mm Hg or diastolic blood pressure >95 mm Hg on repeated measurements; regular medications; and acute illnesses, surgical operations, or hospitalizations in the preceding 4 weeks.

All subjects completed a standardized questionnaire assessing Epworth Sleepiness Scale, demographic data, and sleep-related symptoms. After overnight polysomnogram, fasting blood sample was obtained in the morning. Blood samples were sent immediately to the laboratory for the assay of glucose, lipids, and EPC. Blood samples were centrifuged for sera which were stored at −70 °C for subsequent batch analysis of AGE.

The study was approved by the Institutional Review Board of the University of Hong Kong and the Hong Kong Hospital Authority Hong Kong West Cluster. All subjects gave written informed consent.

### In-hospital overnight polysomnography

Recruited subjects underwent an overnight attended 16-channel PSG (Alice 5 Diagnostics System; Respironics, PA, USA), and the PSG recordings were manually scored by a qualified technologist as previously described [15]. Apnea was defined as complete cessation of airflow for at least 10 s while hypopnea was defined as at least 30 % reduction in airflow that was associated with at least 4 % drop in oxygen saturation. OSA was defined by apnea–hypopnea index (AHI) ≥5/h. Oxygen desaturation index (ODI) was defined as the number of episodes per hour of oxygen saturation drop of at least 4 %.

### Endothelial progenitor cells assay

Citrated blood was collected from patients and the blood cell composition was measured by Coulter counter (Diff-2R, Beckman Coulter). Then, 100 μL of citrated blood was incubated with monoclonal antibodies against human kinase insert domain receptor (KDR) (1:10, R&D Biosystems; FITC-conjugated), CD133 (1:10, Miltenyi; PE-conjugated), CD34 (1:10, Becton Dickinson; APC-conjugated), and CD45 (1:10, Beckman Coulter; PC7-conjugated) for 30 min. After incubation, blood was lysed and fixed by OptiLyse® C solution (Beckman Coulter). Isotypic IgG antibodies were used as controls. For multicolor compensation during flow cytometry analysis (FACS), the blood sample was also stained with FITC, PE, APC, and PC7-conjugated monoclonal antibodies against human CD45 to correct for interfluorescent interference. FACS analysis was preformed by FC500 flow cytometer (Beckman Coulter) in which 10,000 events of lymphocyte-like cells were counted. Endothelial progenitor cell parameters were expressed as absolute counts of CD34+, CD133+, CD34+/KDR+, and CD133+/KDR+ in total lymphocyte-like population.

### Advanced glycation end-products

Serum AGE was measured by competitive enzyme-linked immunosorbent assay (ELISA) using a well-characterized polyclonal rabbit antisera raised by hyperimmunization against AGE-ribonuclease (AGE-RNase), as previously described [7]. This antisera recognizes, as a predominant epitope, the AGE crosslink, ALI (arginine–lysine imidazole). In brief, 96-well plates were coated with 50 μL per well of AGE-RNase (3.75 μg/mL). Fifty microliters of serum (1:4 dilution) was added, followed by 50 μL of 1:500 diluted anti-AGE antibody. Alkaline phosphate-conjugated anti-rabbit IgG (1:2,000) in dilution buffer was added to each well and incubated for 1 h at 37 °C. After washing, color was developed by the addition of 100 μL pNPP substrate (Sigma-Aldrich, St. Louis, MO, USA). Optical density (OD) at 405 nm was determined by an ELISA reader. Results were calculated as $1-\left[ {{{{\left( {\mathrm{experimental}\,\mathrm{OD} - \mathrm{background}\,\mathrm{OD}} \right)}} \left/ {{\left( {\mathrm{total}\,\mathrm{OD} - \mathrm{background}\,\mathrm{OD}} \right)}} \right.}} \right]$. In our assay, 1 U of AGEs is defined as the amount of AGE present in 1:4 diluted serum that causes 50 % competitive inhibition of anti-AGE antibody binding to coated AGE-RNase on the ELISA plate.

### Statistics

Continuous data in normal distribution were presented as mean ± standard deviation, or median and interquartile range otherwise. Categoric variables were presented as percentages. Variables not in normal distribution, including AHI, ODI, duration of oxygen saturation <90 %, arousal index, CD133+ count, and CD34+KDR+ count were natural log-transformed and parametric statistical tests were used for analysis. Partial correlation analysis was used to explore the association between EPC parameters, sleep parameters, and AGE levels, with adjustments for known confounding variables including age, impaired fasting glucose status (fasting glucose ≥ 5.6 or <5.6 mmol/L) [16], overweight status (body mass index ≤ 25 or >25 kg/m^2^), and smoking status (current smoker or noncurrent smoker). Stepwise multivariate linear regression analysis using AGE as the dependent variable was used to investigate its relationship with independent variables as listed in Table 2. SPSS 15.0 software was used for the statistical analysis and a *p* value of <0.05 was considered as statistically significant.

## Results

Eighty-five Chinese subjects were recruited according to inclusion and exclusion criteria. Subsequently, those with extreme obesity (BMI ≥ 35 kg/m^2^, *n* = 3) or grossly elevated low density lipoproteins (LDL ≥ 4.1 mmol/L, *n* = 10) were also excluded, as these factors have been reported to significantly affect EPC levels [16] or commonly warrant drug treatment [17, 18]. Seventy-two subjects (57 men and 15 women) were analyzed. The characteristics of these subjects were listed in Table 1.

**Table 1:** Characteristics of recruited subjects

	Overall (*n* = 72)
Age (years)	43.0 (10.9)
Body mass index (kg/m^2^)	26.1 (3.6)
Smoking status	
Active smoker	19.4 %
Ex-smoker or nonsmoker	80.6 %
Systolic blood pressure (mm Hg)	119.1 (14.0)
Diastolic blood pressure (mm Hg)	71.9 (9.0)
Total cholesterol (mmol/L)	4.8 (0.7)
Triglycerides (mmol/L)	1.5 (1.1, 2.0)
Low density lipoprotein (mmol/L)	2.8 (0.6)
Fasting glucose (mmol/L)	5.1 (0.6)
Epworth Sleepiness Scale	11 (7, 14)
Sleep efficiency (%)	79.1 (12.0)
Apnea–hypopnea index (events/h)	7.6 (2.2, 23.4)
Duration of oxygen desaturation <90 % (min)	3.4 (0.1, 24.4)
Oxygen desaturation index (events/h)	6.3 (1.6, 19.2)
Minimal oxygen saturation (%)	80.1 (12.8)
Arousal index (events/h)	14.3 (9.0, 25.1)
Advanced glycation end-products (U/mL)	3.42 (0.51)
CD34+ (cells/μL)	24.9 (10.3)
CD133+ (cells/μL)	11.9 (6.0, 19.2)
CD34 + KDR + (cells/μL)	4.0 (2.5, 8.5)
CD133 + KDR + (cells/μL)	5.0 (3.1, 9.0)

Variables in normal distribution are expressed as mean ± standard deviation, or median and interquartile ranges for those not in normal distribution

Among various EPC parameters, CD34+ cell counts correlated significantly with the severity of nocturnal hypoxemia. Adjusting for age, smoking status, fasting glucose, and weight status as listed above, circulating CD34+ cell counts correlated positively with ODI (*r* = 0.250, *p* = 0.041) and duration of oxygen desaturation <90 % (*r* = 0.261, *p* = 0.033) and negatively with minimum oxygen saturation (*r* = −0.247, *p* = 0.044). There was no significant correlation of CD34+ cell counts with AHI (*r* = 0.170, *p* = 0.168) or arousal index (*r* = 0.223, *p* = 0.07). CD133+ cells and CD133+/KDR+ cells were both positively correlated with arousal index (*r* = 0.242, *p* = 0.048; *r* = 0.291, *p* = 0.017, respectively) but not with other sleep parameters (Table 2).

**Table 2:** Association between sleep parameters, endothelial progenitor cells, and advanced glycation end-products

*p* values (correlation coefficient)	ODI	T90	Min SaO_2_	Arousal index	AHI	AGE
CD34+ count	0.041 (0.250)	0.033 (0.261)	0.044 (−0.247)	NS	NS	NS
CD133+ count	NS	NS	NS	0.048 (0.242)	NS	0.021 (−0.281)
CD133 + KDR + count	NS	NS	NS	0.017 (0.291)	NS	NS

Number in bracket = correlation coefficient, *AHI* apnea–hypopnea index, *ODI* oxygen desaturation index, *T90* time with oxygen saturation <90 %, *AGE* advanced glycation end-products, *Min SaO*_*2*_ minimal oxygen saturation

AGE levels significantly correlated with indices of OSA severity after adjustment for confounding factors: AHI (*r* = 0.249, *p* = 0.042), ODI (*r* = 0.244, *p* = 0.047), duration of oxygen desaturation < 90 % (*r* = 0.247, *p* = 0.043), and minimum oxygen saturation (*r* = −0.251, *p* = 0.041). Among various EPC subpopulations, the number of 133+ cells correlated negatively with AGE levels (*r* = −0.281, *p* = 0.021).

To further analyze the association between EPC, AGE, and sleep parameters, a stepwise multiple linear regression analysis was performed with AGE as dependent variables. Minimal oxygen saturation (*p* = 0.013) and CD133+ counts (*p* = 0.029) were found to be significant independent determinants of AGE level (*R* ^2^ = 0.147) (Table 3).

**Table 3:** Stepwise multiple linear regression model of advanced glycation end-products

Adjusted *R* ^2^ = 14.7 %			
	Adjusted *R* ^2^	Coefficient beta	*p* value
Minimal oxygen saturation^a^	7.1 %	−0.287	0.013
CD133+ counts^a^	5.1 %	−0.250	0.029

Independent variables included age, current smoking status, overweight status, low density lipoprotein, impaired fasting glucose, apnea–hypopnea index^a^, oxygen desaturation index^a^, duration of oxygen desaturation <90 %^a^, arousal index^a^, minimum oxygen saturation, CD133+ counts^a^, CD34+ counts, CD 34 + KDR + counts^a^, CD 133 + KDR + counts^a^

^a^Log transformation before analysis

## Discussion

The current study shows the alteration in EPC profile in relation to indices of OSA severity and levels of AGE, in subjects free of adverse clinical metabolic parameters and overt cardiovascular diseases. Circulating CD34+ cell count increased with severity of intermittent hypoxia, while CD133+ cell count diminished in correlation with higher AGE levels.

Circulating EPC, which are thought to originate from myeloid pluripotent stem cells, represent the capacity for endothelial regeneration and neovascularization [11, 19]. After being released from bone marrow, they undergo further differentiation with a sequence of changes in their surface antigen (cluster of differentiation, CD), the pattern of which forms the basis of identifying circulating EPC by fluorescence activated cell sorting analysis [20]. CD133+ cells represent the pool of early pluripotent stem cells, which mobilize from bone marrow and differentiate by losing the surface marker and acquiring other markers indicating maturation such as CD34 and/or KDR [13]. CD34+ progenitor cells, sometimes being regarded as late EPC [12], represent more mature endothelial-like cells, which have high angiogenic potential and show the best correlation with cardiovascular risk estimates [21]. Baseline EPC is reduced in the number or becomes dysfunctional in chronic conditions such as aging, hypertension, diabetes, hypercholesterolemia, smoking, and obesity [13]. On the other hand, in response to acute tissue hypoxia such as acute myocardial infarction or unstable angina, there is a physiological surge in circulating EPC levels and homing to damaged sites in order to facilitate repair sequences and new vessel formation in both human and murine studies [22–24]. Advanced glycation end-products have been reported to correlate with reduced number of EPC (CD133+KDR+, CD34+KDR+) in a group of healthy volunteers [6] and to promote EPC dysfunction and apoptosis in vitro [25].

Several previous studies have investigated on the association between OSA and EPC. Findings have been inconclusive, with both negative [26, 27] and positive studies [28–31]. An American study [30] found a lower baseline flow-mediated dilation (FMD) and EPC (CD34+CD133+KDR+) in OSA patients (*n* = 22) when compared to control subjects (*n* = 15), and EPC decreased across tertiles of increasing severity of OSA. Both FMD and EPC increased significantly after CPAP therapy for 4 weeks, while this was not observed in those noncompliant to CPAP therapy. A Japanese case–control study has nicely shown that several biomarkers of ischemia-induced angiogenesis or oxidative stress, including angiotensin, vascular endothelial growth factor, oxidized low density lipoprotein, and circulating EPC (CD34+CD133+CD202b+CD45−), were increased in the OSA group and decreased after nasal CPAP treatment [28]. A Spanish study also adopted a case–control model and defined EPC as CD34+VEGFR-2+. The authors found a lower EPC level in OSA patients (*n* = 13) compared to healthy control (*n* = 13), but no significant correlation was found between EPC and AHI, and the effect of intermittent hypoxia was not reported [29]. On the contrary, another case–control study found no difference in the quantity of CD34+CD133+ EPC between OSA subjects (*n* = 17) and controls (*n* = 10). In a recent pediatric study, EPC counts (CD34+KDR+VEGFR2+) were reduced concordantly with impaired endothelial function in prepubertal children with OSA compared to control subjects [32], and the difference EPC density appeared to account at least partly to the variation in endothelial functional phenotypes in those with OSA. A study from Korea investigated endothelial colony forming units (CFU) of cultured EPC and found similar levels of CFU in samples from OSA subjects (*n* = 82) compared to controls (*n* = 22), and no correlation between severity of OSA and number of CFU could be identified [27].

In the present study, the increase in CD34+ cells correlating with the severity of nocturnal intermittent hypoxemia is consistent with mobilization of EPC in response to acute hypoxia–reoxygenation injury in these subjects relatively free of conventional cardiovascular risk factors or disease. On the other hand, CD133+ population, which represents immature progenitor cells, was reduced in correlation with higher AGE levels, suggesting that the accumulation of AGE in OSA subjects whose condition remain untreated for a long time could jeopardize the EPC pool and may forebode impairment of vascular repair capacity and endothelial dysfunction. The heterogeneity in findings among various studies could be explained by the adoption of different definitions and quantification methods for EPC populations, and the varying stringency in exclusion of confounding vascular co-morbidities. In the current study, meticulous effort was made to exclude potential confounding risk factors. Polysomnography was manually scored by one dedicated technician not involved in other parts of this study. A larger sample size might allow more reliable analysis and interpretation.

Our findings allow further understanding of vascular pathogenesis in OSA involving EPC and AGE. Reduced ability to trigger endothelial repair mechanisms in response to hypoxic tissue injury can occur at later time points of the natural course of OSA. Further rigorous clinical trials exploring the therapeutic effect of CPAP are awaited.
